# Supplementary material for: The Evolution and Characterization of the RNA Interference Pathways in Lophotrochozoa
Source: Genome Biol Evol. 2024 May 7;16(5):evae098. doi: 10.1093/gbe/evae098 (PMC11114477; doi:10.1093/gbe/evae098)
Supplement: evae098_Supplementary_Data [file evae098_supplementary_data.zip › Supplementary_materials.zip!/selected_miRNAs/NoGe_selected_miRNAs/mirdeep_pdf_files/NMRB01000087.1_Notospermus_geniculatus_strain_Ushimado_scaffold87,_whole_genome_shotgun_sequence_3154.pdf]

Provisional ID : NMRB01000087.1_Notospermus_geniculatus_strain_Ushimado_scaffold87,_whole_genome_shotgun_sequence_3154

Score total	:	2852.2
Score for star read(s)	:	3.9
Score for read counts	:	2843.4
Score for mfe	:	1.8
Score for randfold	:	0
Score for cons. seed	:	3
Total read count	:	5589
Mature read count	:	5583
Loop read count	:	0
Star read count	:	6

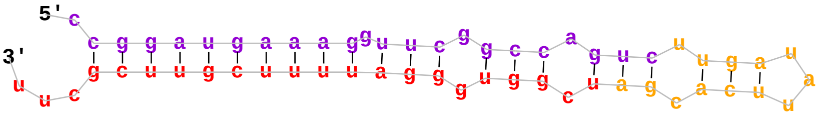

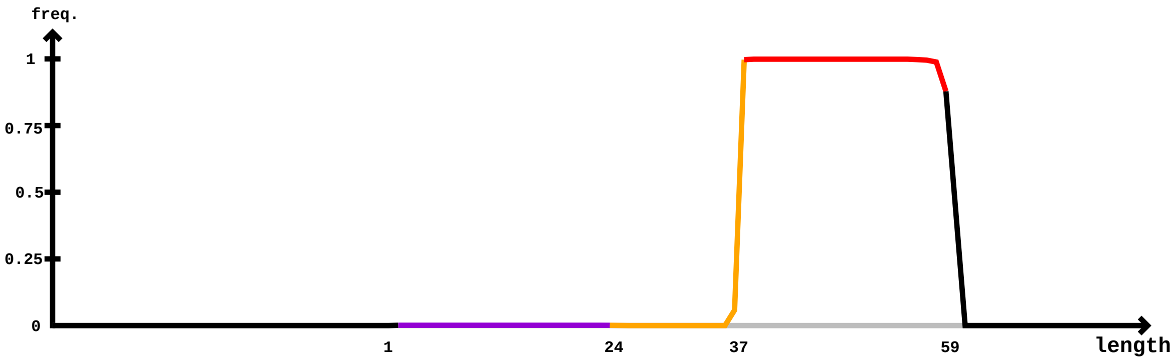

Star Mature

```
5'- cucaaauaaggauggugagcgcuacuuguuucguccggaugaaagguucggccagucuugauauucacgaucggugggauuuucguucgcuuggaauaaguagcaguccuuc  -3'   obs
    cucaaauaaggauggugagcgcuacuuguuucguccggaugaaagguucggccagucuugauauucacgaucggugggauuuucguucgcuuggaauaaguagcaguccuuc         exp
    .......((((((.......((((((((((((..(((((((((.(((.(((.(((.(((...))).))).))).))).)))))))))))))..))))))))))))).)))))).   reads   mm   sample
    .....................................................................aucggugggauuuucguucgc......................   1       0    Mm3
    .....................................................................aucggugUgauuuucguucgcu.....................   1       1    Mm3
    .....................................................................aucggugggauuuucguAcgcu.....................   1       1    Mm3
    .....................................................................aucggugggauuuucguucgcu.....................   30      0    Mm3
    .....................................................................Cucggugggauuuucguucgcuu....................   1       1    Mm3
    .....................................................................aucggugggauuuucguucgcuu....................   4       0    Mm3
    .....................................................................aucggugggauuuucguucgcuuU...................   1       1    Mm3
    ......................................................................ucggugggauuuucguuc........................   1       0    Mm3
    ......................................................................ucggugggauuuucguucU.......................   1       1    Mm3
    ......................................................................ucggugggauuuucguucg.......................   2       0    Mm3
    ......................................................................ucggugggauuuucguucgc......................   7       0    Mm3
    ......................................................................ucggugggauuuucguuGgcu.....................   1       1    Mm3
    ......................................................................ucggugggauuuucguuAgcu.....................   1       1    Mm3
    ......................................................................ucUgugggauuuucguucgcu.....................   2       1    Mm3
    ......................................................................ucggugggauuuucguucgcu.....................   57      0    Mm3
    ......................................................................ucggugggauuuucguucgcG.....................   1       1    Mm3
    ......................................................................ucggugggauuCucguucgcuu....................   1       1    Mm3
    ......................................................................ucggugggauAuucguucgcuu....................   1       1    Mm3
    ......................................................................ucggugggauuuucguucgcuA....................   1       1    Mm3
    ......................................................................ucggugggauuuucguCcgcuu....................   2       1    Mm3
    ......................................................................ucggugggauuuucguucgcuC....................   1       1    Mm3
    ......................................................................ucggugggaGuuucguucgcuu....................   1       1    Mm3
    ......................................................................uAggugggauuuucguucgcuu....................   1       1    Mm3
    ......................................................................ucAgugggauuuucguucgcuu....................   1       1    Mm3
    ......................................................................ucggugggauuuucguucgcuG....................   5       1    Mm3
    ......................................................................ucggugggauuuucguuUgcuu....................   3       1    Mm3
    ......................................................................ucggugggauGuucguucgcuu....................   1       1    Mm3
    ......................................................................ucggugCgauuuucguucgcuu....................   1       1    Mm3
    ......................................................................ucggugggauuuucguuGgcuu....................   2       1    Mm3
    ......................................................................ucggugggauuuucguuAgcuu....................   6       1    Mm3
    ......................................................................ucggugggauuuucguGcgcuu....................   2       1    Mm3
    ......................................................................ucggugggauuuucguAcgcuu....................   2       1    Mm3
    ......................................................................ucggugggauuuucguucgcuu....................   692     0    Mm3
```

```
                                  Star                              Mature
cucaaauaaggauggugagcgcuacuuguuucguccggaugaaagguucggccagucuugauauucacgaucggugggauuuucguucgcuuggaauaaguagcaguccuuc
......................................................................ucggugggauuuucguucgcuuC...................   1     1   Mm3
......................................................................ucggugggauuuucguucgcuuU...................   220   1   Mm3
......................................................................ucggugggauuuucguucgcuuA...................   8     1   Mm3

.....................................................................aucggugggauuuucguGcgc......................   1     1   Fm3
.....................................................................aucggugggauuuucguucgc......................   6     0   Fm3
.....................................................................aucggugggauuuucguAcgcu.....................   2     1   Fm3
.....................................................................aucggugggauuuucguucgcu.....................   144   0   Fm3
.....................................................................Uucggugggauuuucguucgcu.....................   1     1   Fm3
.....................................................................aucggugggauuuucguucgcG.....................   1     1   Fm3
.....................................................................aucggugggauuuucgAucgcu.....................   1     1   Fm3
.....................................................................aucggugggauuuucgCucgcu.....................   1     1   Fm3
.....................................................................aucggugggauuuucguCcgcu.....................   2     1   Fm3
.....................................................................Nucggugggauuuucguucgcu.....................   1     1   Fm3
.....................................................................aucggugggauuuucguucgcuu....................   14    0   Fm3
.....................................................................aucggugggauuuucguucgcuuU...................   1     1   Fm3
......................................................................ucggugggauuuucguucU.......................   3     1   Fm3
......................................................................ucggugggauuuucguucg.......................   3     0   Fm3
......................................................................ucggugggauuuucguucA.......................   2     1   Fm3
......................................................................ucggugggauuuucguucgc......................   13    0   Fm3
......................................................................ucggugggauuuucguucgU......................   2     1   Fm3
......................................................................ucggugggauuuucguucgcu.....................   135   0   Fm3
......................................................................ucgguggguuuuucguucgcuu....................   1     1   Fm3
......................................................................ucggCgggauuuucguucgcuu....................   1     1   Fm3
......................................................................ucggugggauGuucguucgcuu....................   1     1   Fm3
......................................................................ucggugggauuuucguGcgcuu....................   1     1   Fm3
......................................................................Ccggugggauuuucguucgcuu....................   1     1   Fm3
......................................................................ucggugggauuuucguuUgcuu....................   4     1   Fm3
......................................................................ucggugggauuuucguucUcuu....................   2     1   Fm3
......................................................................ucggugggauuuucguAcgcuu....................   3     1   Fm3
......................................................................ucggugggauAuucguucgcuu....................   3     1   Fm3
......................................................................ucggugggauuuucguuGgcuu....................   4     1   Fm3
......................................................................ucggugggauuuucguucgcCu....................   1     1   Fm3
......................................................................ucggGgggauuuucguucgcuu....................   1     1   Fm3
......................................................................ucggugggauuuucguuAgcuu....................   5     1   Fm3
......................................................................ucggugggauuuucguCcgcuu....................   3     1   Fm3
......................................................................ucggugggauuuucgCucgcuu....................   1     1   Fm3
......................................................................ucggugggauuuucguucgcuu....................   849   0   Fm3
......................................................................ucggugggauuuucguucgcuA....................   4     1   Fm3
......................................................................ucggugggauuuucguucgcuuU...................   23    1   Fm3
......................................................................ucggugggauuuucguucgcuuA...................   1     1   Fm3
.......................................................................cggugggauuuucguucgcuu....................   3     0   Fm3

..................................ccggaugaaagguucggccagu........................................................   1     0   Mm2
..................................ccUgaugaaagguucggccaguc.......................................................   1     1   Mm2
.....................................................................aucggugggauuuucguucgc......................   1     0   Mm2
.....................................................................aucggugggauuuucguucgcG.....................   1     1   Mm2
.....................................................................aucggugggauuuucguucgcu.....................   20    0   Mm2
.....................................................................aucggugggauuuucguAcgcu.....................   1     1   Mm2
.....................................................................aucggugggauuuucguucgcuu....................   1     0   Mm2
.....................................................................aucggugggauuuucguucgcuuU...................   1     1   Mm2
......................................................................ucggugggauuuucguuc........................   1     0   Mm2
......................................................................ucggugggauuuucguucgc......................   2     0   Mm2
......................................................................ucggugggauuuucguucgcG.....................   2     1   Mm2
......................................................................ucggugggauAuucguucgcu.....................   1     1   Mm2
......................................................................ucggugggauuuucguGcgcu.....................   1     1   Mm2
......................................................................ucggugggauuuucguucgcu.....................   63    0   Mm2
......................................................................ucggugggauuuucguuUgcu.....................   1     1   Mm2
......................................................................ucggugggauuuucgCucgcu.....................   1     1   Mm2
......................................................................ucggugggauCuucguucgcuu....................   1     1   Mm2
......................................................................ucggugggGuuuucguucgcuu....................   1     1   Mm2
......................................................................ucgguAggauuuucguucgcuu....................   1     1   Mm2
......................................................................ucggugggauuuucguCcgcuu....................   5     1   Mm2
......................................................................ucggCgggauuuucguucgcuu....................   1     1   Mm2
......................................................................ucggugggauuuucguucgcuG....................   7     1   Mm2
......................................................................ucggugggauuuucguucgcuu....................   814   0   Mm2
......................................................................ucUgugggauuuucguucgcuu....................   1     1   Mm2
......................................................................ucggugggauuuucguAcgcuu....................   2     1   Mm2
......................................................................ucggugggauuuucguGcgcuu....................   3     1   Mm2
......................................................................ucggugggauuuucguuUgcuu....................   8     1   Mm2
```

```
                                  Star                            Mature
cucaaauaaggauggugagcgcuacuuguuucguccggaugaaagguucggccagucuugauauucacgaucggugggauuuucguucgcuuggaauaaguagcaguccuuc
......................................................................Ncggugggauuuucguucgcuu....................    1    1    Mm2
......................................................................ucggugggauuuucguuAgcuu....................    6    1    Mm2
......................................................................ucggugggauuuuUguucgcuu....................    1    1    Mm2
......................................................................ucggugggauGuucguucgcuu....................    1    1    Mm2
......................................................................ucggugAgauuuucguucgcuu....................    1    1    Mm2
......................................................................ucggugggauuuucguuGgcuu....................    4    1    Mm2
......................................................................ucggugggauuuucguucgcuA....................    1    1    Mm2
......................................................................ucggugggauuuucUuucgcuu....................    1    1    Mm2
......................................................................ucggugggaAuuucguucgcuu....................    1    1    Mm2
......................................................................ucggugggauuuucguucgcuuU...................    259  1    Mm2
......................................................................ucggugggauuuucguucgcuuC...................    2    1    Mm2
......................................................................ucggugggauuuucguucgcuuA...................    8    1    Mm2
......................................................................ucggugggauuuucguucgcuug...................    1    0    Mm2
.......................................................................cggugggauuuucguucgcuu....................    1    0    Mm2
.......................................................................cggugggauuuucguucgcuuU...................    1    1    Mm2

......................................................................ucggugggauuuucguucgc......................    5    0    Mm1
......................................................................ucggugggauuuucguucgcuu....................    100  0    Mm1
......................................................................ucggugggGuuuucguucgcuu....................    1    1    Mm1
......................................................................ucggugggauuuucguuAgcuu....................    1    1    Mm1
......................................................................ucggugggauuuucguuGgcuu....................    1    1    Mm1
......................................................................ucggugggauuuucguucgcuuU...................    16   1    Mm1

..................................ccggaugaaagguucggccaguc.......................................................    4    0    Fm1
.....................................................................aucggugggauuuucguucgcG.....................    1    1    Fm1
.....................................................................aucggugggauuuucguucgcu.....................    21   0    Fm1
.....................................................................aucggugggauuuucguucgcuu....................    1    0    Fm1
.....................................................................Cucggugggauuuucguucgcuu....................    1    1    Fm1
.....................................................................aucggugggauuuucguucgcuuU...................    2    1    Fm1
......................................................................ucggugggauuuucguuA........................    1    1    Fm1
......................................................................ucggugggauuuucguuc........................    6    0    Fm1
......................................................................ucggugggauuuucguucgc......................    2    0    Fm1
......................................................................ucggugggauuuucguucgcu.....................    63   0    Fm1
......................................................................ucggugggauuuucguuUgcu.....................    1    1    Fm1
......................................................................ucggugggauuuucguuAgcu.....................    3    1    Fm1
......................................................................ucggugggauGuucguucgcuu....................    1    1    Fm1
......................................................................ucggugCgauuuucguucgcuu....................    1    1    Fm1
......................................................................ucggugggauuuucguucgcuG....................    1    1    Fm1
......................................................................ucggugggauuuucguucgcGu....................    2    1    Fm1
......................................................................ucggugggauuuuAguucgcuu....................    1    1    Fm1
......................................................................ucggugggauuuuUguucgcuu....................    1    1    Fm1
......................................................................ucggugggauuuucguuUgcuu....................    2    1    Fm1
......................................................................ucggugggauuuucguuAgcuu....................    5    1    Fm1
......................................................................ucggugggauuuucguucgcuA....................    1    1    Fm1
......................................................................Ncggugggauuuucguucgcuu....................    1    1    Fm1
......................................................................ucggugggauuuucguGcgcuu....................    2    1    Fm1
......................................................................ucggugggauuuucguuGgcuu....................    3    1    Fm1
......................................................................ucggugggaGuuucguucgcuu....................    1    1    Fm1
......................................................................ucgguggAauuuucguucgcuu....................    1    1    Fm1
......................................................................ucUgugggauuuucguucgcuu....................    3    1    Fm1
......................................................................ucggugggauuuucguCcgcuu....................    3    1    Fm1
......................................................................ucggugggauuuucguAcgcuu....................    1    1    Fm1
......................................................................ucggugggauuuucguucgcuu....................    680  0    Fm1
......................................................................ucgguggCauuuucguucgcuu....................    1    1    Fm1
......................................................................ucgguNggauuuucguucgcuu....................    1    1    Fm1
......................................................................ucggugggauAuucguucgcuu....................    3    1    Fm1
......................................................................ucggugggauuuucguucgcuuU...................    195  1    Fm1

.....................................................................aucggugggaGuuucguucgcu.....................    1    1    Fm2
.....................................................................aucggugggauuuucguucgcu.....................    31   0    Fm2
.....................................................................aucggugggauuuucguucgcG.....................    1    1    Fm2
.....................................................................aucggugggauuuucguCcgcu.....................    1    1    Fm2
.....................................................................aucggugggauuuucguucgcuu....................    25   0    Fm2
......................................................................ucggugggauuuucguucgcG.....................    1    1    Fm2
......................................................................ucggugggauuuucguCcgcu.....................    1    1    Fm2
......................................................................ucggugggauuuucguucgcu.....................    16   0    Fm2
......................................................................ucggugggauuuucguCcgcuu....................    2    1    Fm2
......................................................................ucggugggauuuucguucgcuu....................    459  0    Fm2
......................................................................ucggugggauuuuAguucgcuu....................    1    1    Fm2
......................................................................ucgguUggauuuucguucgcuu....................    2    1    Fm2
```

```
                                  Star                                 Mature
cucaaauaaggauggugagcgcuacuuguuucguccggaugaaagguucggccagucuugauauucacgaucggugggauuuucguucgcuuggaauaaguagcaguccuuc
......................................................................ucggugggGuuuucguucgcuu....................   1     1    Fm2
......................................................................ucggugggauuuucguuAgcuu....................   1     1    Fm2
......................................................................ucggugggauuuucguGcgcuu....................   2     1    Fm2
......................................................................ucggugggauuuucguuGgcuu....................   1     1    Fm2
......................................................................ucgguAggauuuucguucgcuu....................   1     1    Fm2
......................................................................Ncggugggauuuucguucgcuu....................   1     1    Fm2
......................................................................ucggugggauuuucguuUgcuu....................   3     1    Fm2
......................................................................ucggugggauuuucguucgcuG....................   2     1    Fm2
......................................................................ucggugggauuuucguucgcuA....................   10    1    Fm2
......................................................................ucggugggauGuucguucgcuu....................   1     1    Fm2
......................................................................ucggugggauuuucguucgcuuA...................   31    1    Fm2
......................................................................ucggugggauuuucguucgcuuU...................   307   1    Fm2
.......................................................................cggugggauuuucguucgcuuU...................   6     1    Fm2
```
